# Comparison of Three Lateral Flow Immunoassay Formats for the Detection of Antibodies against the SARS-CoV-2 Antigen

**DOI:** 10.3390/bios13070750

**Published:** 2023-07-20

**Authors:** Dmitriy V. Sotnikov, Nadezhda A. Byzova, Anatoly V. Zherdev, Youchun Xu, Boris B. Dzantiev

**Affiliations:** 1 A.N. Bach Institute of Biochemistry, Research Center of Biotechnology of the Russian Academy of Sciences, Leninsky Prospect 33, 119071 Moscow, Russia; nbyzova@inbi.ras.ru (N.A.B.); zherdev@inbi.ras.ru (A.V.Z.); dzantiev@inbi.ras.ru (B.B.D.); 2 State Key Laboratory of Membrane Biology, Department of Biomedical Engineering, School of Medicine, Tsinghua University, Beijing 100084, China; xyc2012@mail.tsinghua.edu.cn

**Keywords:** immunochromatography, COVID-19, serodiagnostics, immune complexes, serum testing

## Abstract

Reliable detection of specific antibodies against pathogens by lateral flow immunoassay (LFIA) greatly depends on the composition of the detectable complex and the order of its assembly. We compared three LFIA formats for revealing anti-SARS-CoV-2 antibodies in sera with the following detected complexes in the analytical zone of the strip: antigen–antibodies–labeled immunoglobulin-binding protein (Scheme A); antigen–antibodies–labeled antigen (Scheme B); and immunoglobulin-binding protein–antibodies–labeled antigen (Scheme C). The lowest detection limit was observed for Scheme C, and was equal to 10 ng/mL of specific humanized monoclonal antibodies. When working with pooled positive sera, Scheme C had a detection limit 15 times lower than Scheme B and 255 times lower than Scheme A. Due to the high sensitivity of Scheme C, its application for the panel of human sera (n = 22) demonstrated 100% diagnostic specificity and sensitivity. These consistent results be useful for designing the format of LFIA serodiagnosis for other diseases.

## 1. Introduction

Currently, lateral flow immunoassay (LFIA) is widely used in the primary diagnostics of infectious diseases. Its indisputable advantages are feasibility at sampling places, simplicity, rapidity, and, as a result, quick diagnostics. Both the pathogen itself and antibodies to it can be detected using LFIA. Although the generation of antibodies in an organism takes time (for example, IgG molecules appear in blood during the second week after initial infection), the monitoring of antibodies has advantages compared with pathogen detection. Diagnostic conclusions based on the detection of the pathogen or its compounds/metabolites depend on sampling technique and localization of the sampling point since the pathogen is unevenly distributed in the body. Quite often, false-negative test results of such testing are associated precisely with incorrect sampling [1,2]. The humoral immune response leads to an increase in specific antibodies concentration in the bloodstream. Therefore, conventional blood sampling methods are acceptable for all serodiagnostic assays. [3,4,5]. Due to this, LFIA of antibodies (serodiagnostics) is actively used in practice [6,7,8]. In this LFIA, complexes labeled with a colored nanoparticle are formed in the analytical zone if antibodies to a pathogen are present in the sample, and the colored complexes are absent if there are no specific antibodies in the sample. Colored labels in common LFIA tests are gold nanoparticles or latex particles, but tests with other types of labels have also been developed [9,10,11,12].

**Figure 1:**
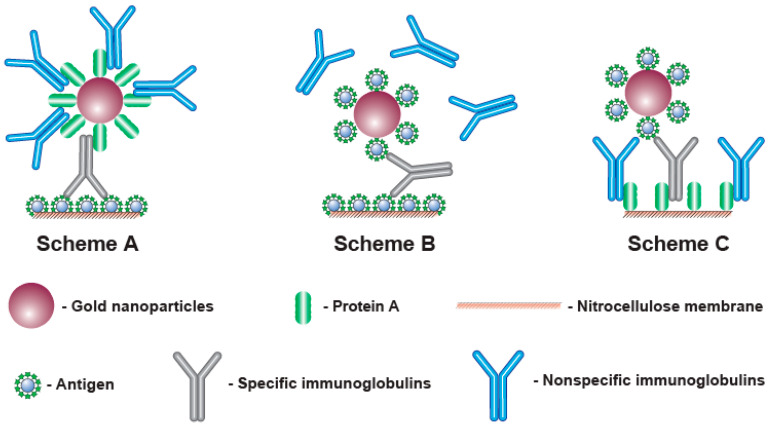
Complexes formed in the analytical zones for three considered schemes of serodiagnostic LFIA (see comments in the text).

To detect antibodies, different formats of LFIA can be implemented that differ in the components of the detected complex and the order of their assembly. The most known approach is to apply an antigen of a given pathogen to the analytical zone, while complexes with an immunoglobulin-binding protein (such as anti-species antibodies, bacterial proteins A, G, L, etc. [13,14,15]) immobilized on a nanoparticle are formed in the fluid flowing along the test strip (Scheme A, see Figure 1, A). However, immunoglobulin-binding proteins interact with all immunoglobulins in the sample, not just with specific antibodies against the given antigen. Considering that specific antibodies to a certain antigen are a small part (a few percent or fractions of a percent) of all immunoglobulins, most of the immunoglobulin-binding proteins are blocked by non-specific immunoglobulins. This blocking reduces the binding of the label in the analytical zone and makes reliable serodiagnostics difficult at low contents of specific antibodies [16,17]. The use of additional stages of the assay to enhance the recorded signal is possible [18,19], but it deprives LFIA of its main advantages in rapidity and easy implementation.

This limitation can be overcome in various ways. Thus, immunoglobulin-binding proteins can be immobilized in the analytical zone, and antigen molecules can be conjugated with a nanoparticle (scheme C—see Figure 1, C) [20,21,22,23]. Since the total surface area of a porous membrane in the analytical zone for sorption is much larger than the achievable total surface of nanoparticles in a colloidal solution moving along the test strip, such a change in the assembly order of the detected complexes reduces the loss of the detected signal.

Another way is to use the polyvalence of antibodies (from 2 for IgG, the most abundant immunoglobulins in the blood, to 10 for IgM). Application of the antigen both to the analytical zone and to the surface of the nanoparticle leads to the formation of complexes detected only by specific antibodies, whereas the rest of the immunoglobulins do not affect the assay results [24,25,26,27,28] (scheme B—see Figure 1, B). However, in this case, signal losses are also possible, since complexes (labeled antigen—antibodies—labeled antigen) can be formed in the flow of liquid along the test strip. As a result, the specific IgG molecules included in these complexes do not have free valences to bind with the antigen in the analytical zone.

As seen from the above, each variant has limitations. Note that serodiagnostics of different diseases have their own features associated with the properties of antigens and different distributions due to the affinity of generated specific antibodies. Therefore, the conclusions about the best format for LFIA serodiagnostics for specific infections can only be made on the basis of experimental comparisons. The existing comparative studies [29,30] demonstrate the limitations of scheme A, but they cover only a few diagnostic systems.

Despite the social significance of COVID-19 diagnostics and the wide range of lateral flow tests developed for this purpose, including commercially available ones, the question of the optimal format for LFIA serodiagnostics of COVID-19, to the best of our knowledge, has not been considered. Such a comparison was the task of this study. The receptor-binding domain (RBD) protein of the SARS-CoV-2 virus, the most popular and informative reagent for diagnosing COVID-19, was used in the work, and recombinant staphylococcal protein A was the reactant for immunoglobulins binding. The study included the choice of optimal conditions for all three variants of LFIA serodiagnostics using these reagents, that is, the concentrations of applied proteins and gold nanoparticle conjugates which provide the maximum specific signal with the minimum non-specific binding. Under the selected conditions, the three variants were quantitatively compared using standard antibody preparations and then tested on a panel of positive and negative sera.

## 2. Materials and Methods

### 2.1. Chemicals and Materials

The recombinant RBD of SARS-CoV-2 spike protein and the monoclonal antibodies to it, clone 5324 (AB_RBD_5324), were from HyTest (Moscow, Russia). According to the manufacturer’s data sheet, these antibodies can recognize Alpha Strain (“British”), Beta Strain (“South African”) and Gamma Strain (“Brazilian”) RBD in addition to wild-type RBD.

The recombinant staphylococcal protein A and the peroxidase-labeled polyclonal goat anti-human antibodies were from Imtek (Moscow, Russia). The goat anti-mouse immunoglobulins (GAMI) and the conjugate of gold nanoparticles and protein A were from Arista Biologicals (Allentown, PA, USA).

Human sera with and without antibodies to SARS-CoV-2 were kindly provided by Dr. S.F. Biketov (State Research Center of Applied Microbiology and Biotechnology, Obolensk, Russia). They were obtained from volunteers and patients after obtaining written informed consent, as specified in previous joint studies [31,32]. Coronavirus infection or its absence was confirmed by PCR tests and clinical observations. To obtain pooled preparations of negative serum, ten sera from donors without symptoms of respiratory diseases and without antibodies against RBD (based on the data from the enzyme immunoassay testing) were mixed.

Bovine serum albumin (BSA), gold chloride (HAuCl_4_), Tris, detergents Tween-20 and Triton X-100, 3,3′,5,5′-tetramethylbenzidine dihydrochloride (TMB), sucrose, sodium citrate, and sodium azide were from Sigma-Aldrich (St. Louis, MO, USA). Other chemicals of analytical grades were from Chimmed (Moscow, Russia). Ultrapure water for syntheses with a resistivity of 18.2 MW was prepared using a Simplicity Milli-Q system (Millipore Corporation, Burlington, MA, USA).

Membranes for preparation of test strips including nitrocellulose working membrane (CNPC-15), glass fiber pad for conjugate (PT-R7), sample pad (GFB-R4), and adsorbent pad (AP045) were from Advanced Microdevices (Ambala Cantt, India). The transparent ELISA microplates were manufactured by Corning Costar (Tewksbury, MA, USA).

### 2.2. Synthesis of GNPs

The synthesis of GNPs employing reduction of HAuCl_4_ by citrate was performed as described by Frens [33] with modifications. A solution of HAuCl_4_ (98.5 mL, 0.01%) was heated to 100 °C, and sodium citrate solution (1.5 mL, 1%) was added. The solution was boiled under vigorous stirring for 30 min and then the cooled to room temperature.

### 2.3. Synthesis of RBD–GNPs Conjugate

The conjugate of RBD with GNPs was synthesized by adsorption immobilization. GNPs (optical density at 520 nm (OD_520_) = 1) with pH 7.5 were added to an RBD solution (pH 7.5) and incubated at room temperature under stirring for 30 min. Then, the solution was adjusted to its final concentration of 0.25% for stabilization of the conjugate. After that, the obtained RBD–GNPs conjugate was separated by 15 min of centrifugation at 20,000× *g* and 4 °C (Allegra 64R, Beckman Coulter, Indianapolis, IN, USA). This was followed by resuspension in 10 mM Tris-HCl, pH 9.0, containing 1% sucrose, 1% BSA, and 0.01% NaN_3_. The obtained conjugates were stored after synthesis in a closed container at 4–6 °C. The properties of the conjugates remained stable for at least 3 months.

### 2.4. Characterization of GNPs and Their Conjugates

Gold nanoparticles and their conjugates were applied to grids (300 mesh, Pelco International; Redding, CA, USA) coated with a poly(vinyl formal) film. After placing on glass, the film was exposed to 0.15% *v*/*v* formvar in chloroform.

The transmission electron microscopy (TEM) images were obtained with a JEM CX-100 microscope (Jeol, Tokyo, Japan) and processed using Image Tool software (UTHSCSA, San Antonio, TX, USA).

### 2.5. Preparation of Test Strips

The reagents listed below (in 0.05 M K-phosphate buffer, pH 7.4, containing 0.1 M NaCl (PBS)) were loaded onto the nitrocellulose membrane by an Image Technology IsoFlow dispenser (Lebanon, NH, USA) creating control and analytical zones (CZ and AZ, respectively) with consumption of 0.12 μL per 1 mm of the membrane:

Scheme A—GAMI (CZ; 0.5 mg/mL) and RBD (AZ; 0.25–1.5 mg/mL);
Scheme B—AB_RBD_5324(CZ; 0.5 mg/mL) and RBD (AZ; 0.25–1.5 mg/mL);
Scheme C—AB_RBD_5324 (CZ; 0.5 mg/mL) and protein A (AZ; 0.25–5.0 mg/mL).

The GNPs conjugates, after the addition of Tween 20 (1% *v*/*v*), were loaded onto the glass fiber pad with a consumption rate of 0.8 μL per 1 mm:

Scheme A—protein A–GNPs (OD_520_ from 1 to 8);
Scheme B and Scheme C—RBD–GNPs (OD_520_ from 1 to 8).

The nitrocellulose membranes and the glass fiber pads were dried for at least 12 h at room temperature. After this, they were formed into sheets including the sample and absorbent pads. The sheets were cut using an automatic Index Cutter-1 guillotine (A-Point Technologies; Gibbstown, NJ, USA). The prepared test strips of 3.5 mm width were stored in hermetically sealed bags with a desiccant at room temperature and no significant changes in their analytical characteristics were observed for at least 6 months.

### 2.6. Lateral Flow Immunoassay Implementation and Its Results Processing

The assay was performed at room temperature as follows:

(1) The test strip was placed horizontally;
(2) A tested sample (60 μL) was applied to the sample pad;
(3) The incubation was carried out for 10 min.

After this, the test strips were scanned and the images were processed by TotalLab TL120 software (Nonlinear Dynamics, Newcastle, UK). This process included finding binding zones, generation of color intensity profiles, background subtraction, and calculation of the integral coloration of the found zones.

Each sample was tested twice. The concentration providing an AZ coloration of more than the average value plus three standard deviations of the coloration intensity for a blank probe was considered as the limit of detection (LOD).

### 2.7. ELISA of Human Sera

RBD (1 μg/mL, in PBS) was added to wells of an ELISA microplate and incubated overnight at 4 °C. After this, the wells were washed four times with PBS and 0.05% *v*/*v* detergent Triton X-100 (PBST) to remove unbound molecules. Thereafter, diluted sera (1:25–1:50,000, in PBST) were added and allowed to incubate for 1 h at 37 °C. The microplate was washed, and diluted peroxidase-labeled anti-human antibodies (1:3000, in PBST) were incubated at the same conditions. After final washing, the catalytic activity of the bound peroxidase was recorded. Namely, the substrate solution (0.4 mM TMB and 3 mM H_2_O_2_ in 40 mM citrate buffer, pH 4.0) was applied and the wells were incubated for 15 min. The reaction was finished by adding 1 M H_2_SO_4_ (*v*/*v* = 1:2), and optical densities at 450 nm were measured (Zenyth 3100 photometer, Anthos Labtec Instruments, Wals, Austria).

## 3. Results

### 3.1. Characterization of GNPs and Their Conjugates

**Figure 2:**
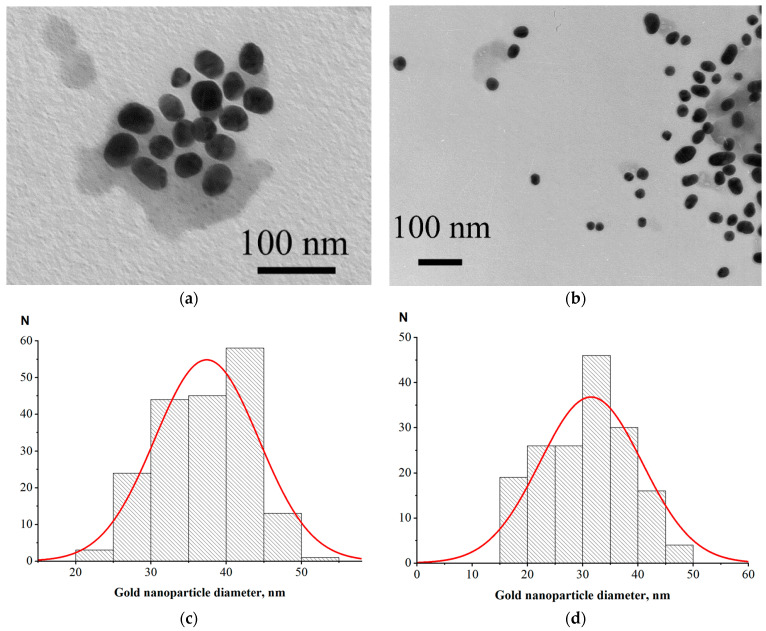
Electron micrographs of GNPs conjugates with RBD (**a**) and staphylococcal protein A (**b**) and histograms of diameter distribution for these conjugates, (**c**) and (**d**), respectively. Red curves are Gaussian fittings of the distributions.

For LFIA according to Scheme A, we used a commercial protein A–GNPs preparation with a declared average nanoparticle diameter of 30 nm. For Schemes B and C, we synthesized GNPs of close size and conjugated them to the RBD antigen. Examples of electron micrographs and histograms of conjugate nanoparticles distributions by diameter (that were built based on microscopy data) are shown in Figure 2. The average nanoparticle diameters were 37 ± 6 nm for RBD–GNPs and 32 ± 9 nm for protein A–GNPs.

### 3.2. Choice of Serum Dilution

To correctly compare LFIA schemes, it is necessary to ensure that the assays are carried out under optimal conditions for each of them, since otherwise the disadvantages of a certain scheme may be due to incorrectly chosen conditions. The key parameters affecting antibody detection in serodiagnostic LFIAs are the concentration of labeled reagent, the concentration of the binding reagent in the AZ, and the serum dilution [16]. Therefore, the LFIAs were optimized for these three parameters.

**Figure 3:**
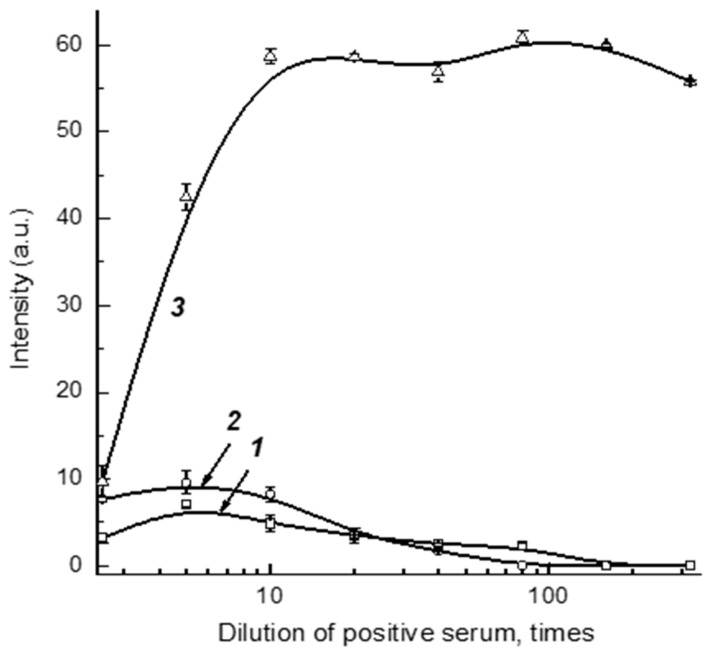
Dependences of coloration intensity of AZ on the dilution of positive pooled serum for test strips that were made according to Schemes A (1), B (2), and C (3).

Dilution of serum samples simultaneously leads to a decrease in the concentrations of controlled specific antibodies and non-specific immunoglobulins that influence the registered signal. Thus, some optimum degree of serum dilution provides the most reliable detection of specific antibodies. To find this value, we used a positive sample prepared from a negative pooled serum and added monoclonal anti-RBD antibodies with a final concentration of 5 μg/mL. The tests of varied dilutions of this preparation by Schemes A, B, and C are presented in Figure 3. As can be seen, the optimal dilutions are somewhat different, but are located in the range 1:5–1:30. With this in mind, the dilution 1:30 was fixed to further investigate the optimal reactant concentrations (see Section 3.3Section 3.4 and Section 3.5), and the final determination of the dilution suitable for work with clinical samples was then performed under optimized concentrations of immunoreactants (see Section 3.7).

Note that the obtained data about serum dilution accord with our earlier investigations for COVID-19 serodiagnostics with Scheme A. We have shown experimentally that anti-RBD antibodies in serum were most reliably detected after a 30-fold dilution, and this result is in accordance with the calculations based on the expected range of concentrations for specific antibodies in positive serum samples [32].

### 3.3. Optimization of LFIA Conditions According to Scheme A

**Figure 4:**
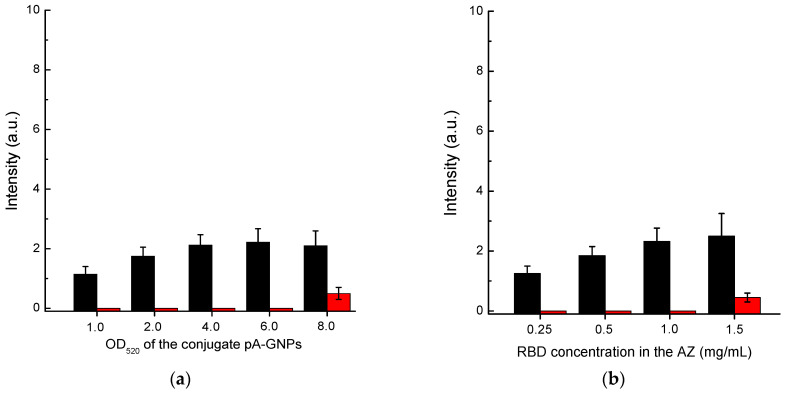
Comparison of conditions for Scheme A of SARS-CoV-2 serodiagnosis: (**a**) choice of OD_520_ for protein A–GNPs conjugate at RBD concentration applied to AZ of 0.5 mg/mL; and (**b**) choice of RBD concentration loaded to AZ at OD_520_ of protein A–GNPs conjugate of 5.0. The red bars in (**a**,**b**) correspond to the 30-fold diluted pooled negative serum, and the black bars correspond to the same diluted serum with added AB_RBD_5324 antibody at a concentration of 5 µg/mL.

First, we optimized the concentration of the protein A–GNPs conjugate. According to the data presented in Figure 4a, the optimal concentration of this conjugate corresponds to its OD_520_ in the range of 4–6. In the OD520 interval from 4 to 8, the intensity of specific coloration showed almost no change, while for OD_520_ > 6, the nonspecific background coloration increased. Therefore, we have chosen the concentration of the protein A–GNPs conjugate corresponding to OD_520_ = 5 as the optimal value.

The RBD concentration in the solution for immobilization varied from 0.25 to 1.5 mg/mL. The results presented in Figure 4b show that the specific coloration of AZ increases over the entire range. However, at a concentration of more than 1 mg/mL, the background coloration increased. Therefore, a concentration of RBD of 1 mg/mL was identified as optimal.

### 3.4. Optimization of LFIA Conditions According to Scheme B

**Figure 5:**
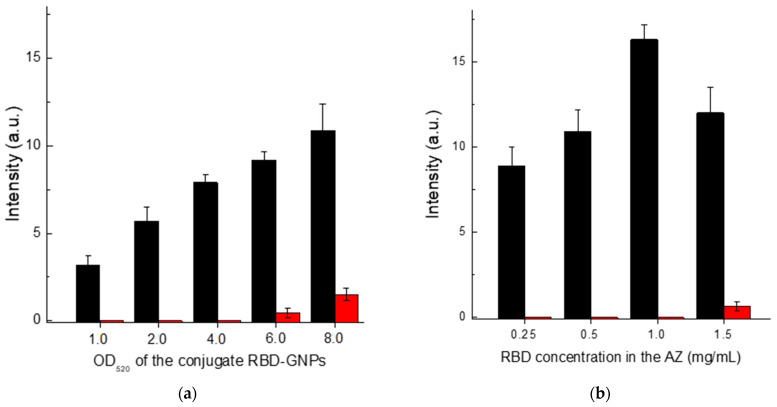
Comparison of conditions for Scheme B of SARS-CoV-2 serodiagnosis: (**a**) choice of OD_520_ for RBD–GNPs conjugate at RBD concentration applied to AZ of 0.5 mg/mL; and (**b**) choice of RBD concentration loaded to AZ at OD_520_ of RBD–GNPs conjugate of 5.0. The red bars in (**a**,**b**) correspond to the 30-fold diluted pooled negative serum, and the black bars correspond to the same diluted serum with added AB_RBD_5324 antibody at a concentration of 5 µg/mL.

Scheme B showed a monotonic increase in the registered coloration of the analytical zone with increasing concentration of the RBD-GNPs conjugate (Figure 5a). However, when the OD_520_ of the GNP-labeled RBD reached 6 or higher, non-specific coloration began to develop. Therefore, the chosen optimum concentration corresponded to OD_520_ = 5.

When varying the RBD concentration in the solution for its immobilization from 0.25 to 1.5 mg/mL, the obtained dependence of the analytical zone coloration had a maximum at 1 mg/mL (Figure 5b). The nonmonotonic dependence is probably associated with polyvalent interactions of immunoglobulins. A RBD concentration of 1 mg/mL also provided a low level of background coloration of the analytical zone and so was used in the final test system completion.

### 3.5. Optimization of LFIA Conditions According to Scheme C

**Figure 6:**
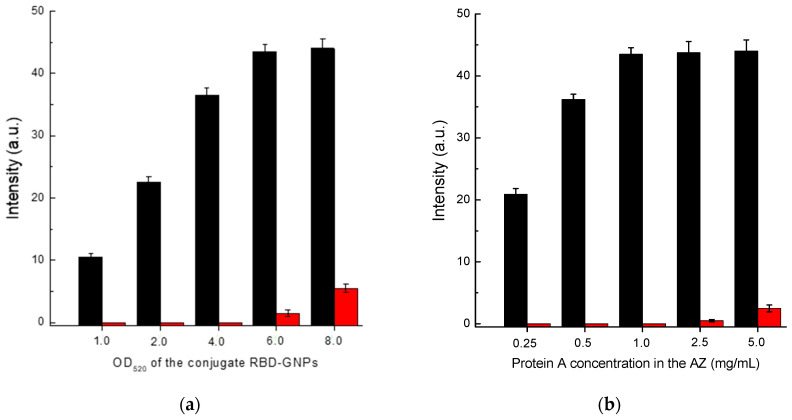
Comparison of conditions for Scheme C of SARS-CoV-2 serodiagnosis: (**a**) choice of OD_520_ for RBD–GNPs conjugate at protein A concentration applied to AZ of 0.5 mg/mL; and (**b**) choice of protein A concentration loaded to AZ at OD_520_ of RBD–GNPs conjugate of 5.0. The red bars in (**a**,**b**) correspond to the 30-fold diluted pooled negative serum, and the black bars correspond to the same diluted serum with added AB_RBD_5324 antibody at a concentration of 5 µg/mL.

In the case of Scheme C, the concentrations of RBD–GNPs conjugate and protein A solution used for immobilization of the analytical zone were optimized. Based on the data presented in Figure 6, the following values of these parameters were chosen: RBD–GNPs conjugate—OD_520_ = 5; protein A concentration applied in the analytical zone—1 mg/mL.

Note that the chosen values of optimal concentrations for the used GNPs conjugates and proteins applied to the analytical zone turned out to be equal for all three schemes of LFIA serodiagnostics: OD_520_ = 5.0, protein concentrations—1 mg/mL.

### 3.6. Comparison of Three Schemes of LFIA Serodiagnostics of SARS-CoV-2 Using Standard Antibody Preparation

**Figure 7:**
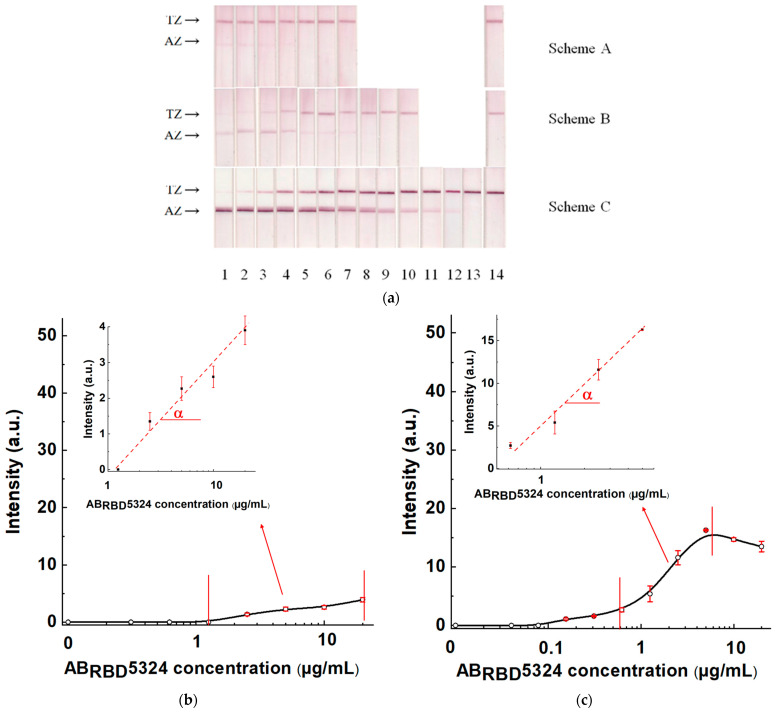

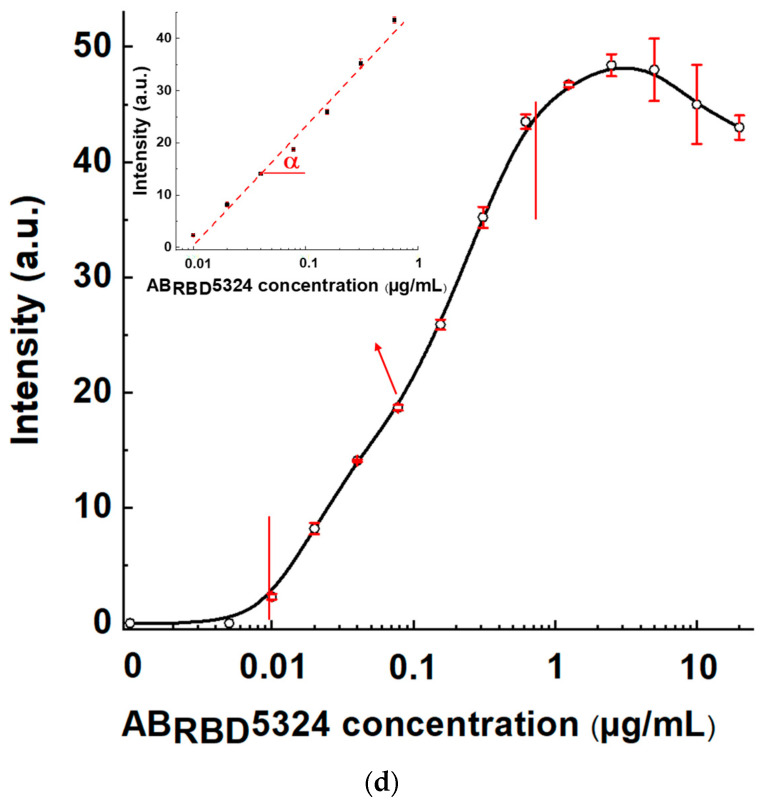
LFIAs of the AB_RBD_5324 antibodies added to the 30-fold diluted pooled negative serum. (**a**) The appearance of test strips after analysis. The AB_RBD_5324 concentrations are 20 (1), 10 (2), 5.0 (3), 2.5 (4), 1.25 (5), 0.62 (6), 0.31 (7), 0.155 (8), 0.078 (9), 0.04 (10), 0.02 (11), 0.01 (12), 0.005 (13) and 0 (14) µg/mL. (**b**–**d**) Calibration curves of Schemes A, B and C, respectively (with added zooms of their working ranges and the indicated slope α).

The test systems manufactured following Schemes A, B, and C and the established optimal concentrations of reagents were used to detect specific antibodies in serum samples. The AB_RBD_5324 antibody was the detectable analyte, and the 30-fold diluted pooled negative serum was the matrix. The results of testing serum samples with various concentrations of the anti-RBD specific antibodies, calibration curves, and their parameters are shown in Figure 7 and Table 1.

For Scheme A, the LOD of antibodies in serum was 2.5 μg/mL, and the maximal reached signal was 4.4 arb. units. For Scheme B, these parameters were 0.155 μg/mL and 16 arb. units, respectively, and for Scheme C—0.01 µg/mL and 48 arb. units, respectively. That is, Scheme A was identified as the the least effective, and Scheme C as the most effective.

### 3.7. Testing of Three LFIA Schemes on Sera from Patients with SARS-CoV-2 and Healthy Persons

Checking the reliability of diagnosis using the three LFIA schemes was carried out by testing the sera of patients. A total of 22 sera were tested, of which 14 belonged to sick patients and 8 to donors without symptoms of respiratory disease (see Table S1 in the Supplement with data of ELISA testing for these sera). According to the ELISA results, the sera were numbered as follows: No. 1–4—strongly positive, No. 5–14—weakly positive, and No. 15–22—negative.

The formed panel of samples was tested in three variants of LFIA for three sera dilutions: 1:10, 1:30, and 1:100. Each sample was tested twice. The full panel of testing results is shown in Table S2 in the Supplement. In terms of signal-to-noise ratio, the most reliable results were obtained when testing sera diluted 30 times. Table 2 integrates data for serum testing under this dilution. A coloration intensity of the analytical zone of 2 arb. units was used as a threshold for distinguishing between positive and negative results. According to our previous experience of using this protocol for digital image processing [32], it corresponds to the threshold for reliable visual detection of coloration.

Schemes A and B showed low efficiency: out of 14 positive sera, only strongly positive ones demonstrated coloration in the analytical zone for both schemes. The coloration intensity for weakly positive samples was near the limit of visual detection, and only two positive results of Scheme A were added following the stated threshold level. The diagnostic sensitivity of Schemes A and B for the used set of samples was lower than 50%. Only for Scheme C was an unambiguous coloration of AZ observed for all positive samples, ensuring a significant difference from negative samples. The diagnostic sensitivity of Scheme C for the used set of samples was 100%. There were no false positive results when testing negative sera for any of the schemes, which corresponds to 100% diagnostic specificity for the used set of samples.

### 3.8. Comparative Evaluation of Three LFIA Schemes

The low efficiency of Scheme A can be explained by the blocking of labeled protein A by non-specific immunoglobulins contained in the samples. The interaction between protein A and non-specific immunoglobulins also occurs in Scheme C but leads to other results. The high efficiency of Scheme C is most likely associated with differences in the sorption capacity of GNPs and the working membrane. GNPs with a diameter of about 30 nm at OD_520_ = 5 are able to bind several micrograms of protein per milliliter (no more than 6 μg for IgG in monolayer immobilization) [34]. This quantity is at least 1000 times less than the average content of IgG per milliliter of blood. Even with a 30-fold dilution of the tested sera, the total content of immunoglobulins remains tens of times higher than the binding capacity of the GNPs conjugate. Thus, in Scheme A, most immunoglobulins are not bound with the label so that most antibodies do not participate in the formation of colored complexes in AZ.

In Scheme C, only specific antibodies bind to the GNP-labeled antigen. The content of these antibodies is orders of magnitude less than the total immunoglobulin fraction of serum. Therefore, there are enough binding sites on the RBD–GNPs conjugate to bind all specific antibodies from the tested sample. In the analytical zone, protein A is immobilized at a concentration of milligrams, which is comparable with the total concentration of immunoglobulins in a diluted serum sample. Therefore, the influence of nonspecific immunoglobulins in Scheme C is weakened, which sharply decreases the limit of antibody detection of Scheme C as compared with Scheme A.

In Scheme B, total immunoglobulins do not affect the assay results, but upon contact with the RBD–GNPs conjugate, specific antibodies can combine RBD–GNPs conjugates into multicompound complexes. Such complexes do not bind to the antigen in the analytical zone due to blocked valencies of specific IgG molecules. In contrast, in Scheme C the antibodies bound to RBD–GNPs conjugates have no restrictions on their interactions with the immobilized protein A in the analytical zone. In addition, different regions of immunoglobulin molecules are involved in interactions with antigen and protein A. Moreover, the complexes with several RBD–GNPs conjugates connected by specific antibodies produce a larger optical signal due to their increased content of GNPs.

## 4. Conclusions

Three LFIA serodiagnostics schemes which differ in the composition and order of formation of the detected complexes were considered: a scheme with labeled protein A and immobilized antigen (Scheme A), a scheme with labeled and immobilized antigens (Scheme B), and a scheme with labeled antigen and immobilized protein A (Scheme C). A comparison of analytical sensitivities for a standard SARS-CoV-2 positive preparation showed that Scheme C had the highest efficiency. The limits of detection for specific antibodies (anti-RBD monoclonal antibody used for spiking) in Schemes A, B, and C were 2.5, 0.155, and 0.01 μg/mL, respectively. In the course of SARS-CoV-2 serodiagnostics for a panel of 14 positive and 8 negative sera, Scheme C showed a two-fold higher diagnostic sensitivity compared with Schemes A and B, whereas diagnostic specificity did not differ for different schemes. The differences in the analytical parameters are probably associated with differences of gold nanoparticles and the working membrane in their sorption capacities, which are reflected in the detection of specific antibodies in tested serum samples in the presence of an excess of nonspecific immunoglobulins.

Due to their low cost, rapidity and applicability in point-of-care conditions, LFIAs seem to be currently the best tools for mass screening diagnostics. However, often these assays are less sensitive compared with laboratory instrumental immunoassays (such as ELISA), which limits field of use of the LFIA technique. This study demonstrates that the analysis scheme with an immobilized immunoglobulin-binding protein and a labeled antigen can reach an LOD close to that of ELISA. These results indicate the competitive advantages of the proposed scheme and the feasibility of introducing systems based on this principle into practice.

## Figures and Tables

**Table 1:** Comparison of SARS-CoV-2 serodiagnostic schemes for anti-RBD antibody detection.

	Limit of Detection, µg/mL	Working Range, µg/mL	Slope α Value, mL/µg
Scheme A	2.5	2.5–20	0.14
Scheme B	0.155	0.8–4.0	3.11
Scheme C	0.01	0.02–0.62	67.54

**Table 2:** Evaluation of the diagnostic efficiency of three SARS-CoV-2 serodiagnostic regimens.

	Confirmed Positive	False Positive	Confirmed Negative	False Negative
Scheme A	6	0	8	8
Scheme B	4	0	8	10
Scheme C	14	0	8	0

## Data Availability

The data that support the findings of this study are available from the corresponding author upon request.

## Supplementary Materials

The following supporting information can be downloaded at: https://www.mdpi.com/article/10.3390/bios13070750/s1, Table S1: ELISA testing of sera diluted 300 time (each sample measured in duplicate); Table S2: LFIA testing of sera diluted 10, 30 and 100 times with test strips by schemes A, B, and C (each sample was tested with two test strips).
