# Supplementary material for: Comparison of Three Lateral Flow Immunoassay Formats for the Detection of Antibodies against the SARS-CoV-2 Antigen
Source: Biosensors (Basel). 2023 Jul 20;13(7):750. doi: 10.3390/bios13070750 (PMC10376990; doi:10.3390/bios13070750)
Supplement: Supplementary file 1 [file biosensors-13-00750-s001.zip › biosensors-2468344-supplementary.pdf]

Supplementary information

Comparison of three lateral flow immunoassay formats for the detection of antibodies against the SARS-CoV-2 antigen

Dmitriy V. Sotnikov[1]*, Nadezhda A. Byzova[1], Anatoly V. Zherdev[1], Youchun Xu[2], Boris B. Dzantiev[1]

[1] A.N. Bach Institute of Biochemistry, Research Center of Biotechnology of the Russian Academy of Sciences, Leninsky prospect 33, Moscow 119071, Russia; sotnikov-d-i@mail.ru; nbyzova@inbi.ras.ru; zherdev@inbi.ras.ru; dzantiev@inbi.ras.ru

[2] State Key Laboratory of Membrane Biology, Department of Biomedical Engineering, School of Medicine, Tsinghua University, Beijing 100084, China; xyc2012@mail.tsinghua.edu.cn

Table S1. ELISA testing of sera diluted 300 time (each sample measured in duplicate)

Serum number	**1**	**2**	**3**	**4**	**5**	**6**	**7**	**8**
OD_{450}	**0.96 ± 0.035**	**0.915 ± 0.025**	**0.81 ± 0.012**	**0.858 ± 0.029**	**0.223 ± 0.035**	**0.438 ± 0.027**	**0.115 ± 0.011**	**0.338 ± 0.018**
Serum number	**9**	**10**	**11**	**12**	**13**	**14**		
OD_{450}	**0.244 ± 0. 065**	**0.305 ± 0.021**	**0.182 ± 0.012**	**0.378 ± 0.009**	**0.351 ± 0.01**	**0.287 ± 0.008**		
Serum number	15	16	17	18	19	20	21	22
OD_{450}	0.107 ± 0.002	0.098 ± 0.001	0.104 ± 0.001	0.184 ± 0.003	0.075 ± 0.002	0.068 ± 0.001	0.082 ± 0.002	0.102 ± 0.002

Table S2. LFIA testing of sera diluted 10, 30 and 100 times with test strips by schemes A, B, and C (each sample was tested with two test strips)

Serum number	Scheme A dilution			Scheme B dilution			Scheme C dilution		
	1:10	1:30	1:100	1:10	1:30	1:100	1:10	1:30	1:100
1	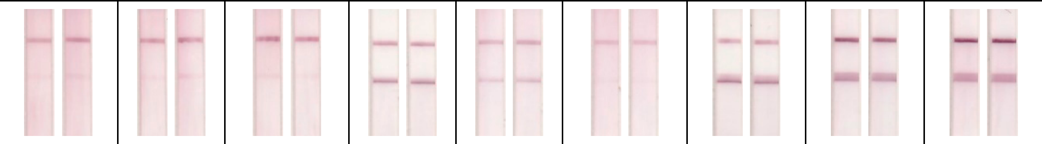								
I (a.u.)	3.2 ± 0.7	4.5 ± 0.75	4.4 ± 0.15	23.0 ± 0.55	10.3 ± 1.1	2.25 ± 0.15	28.5 ± 1.4	27.4 ± 0.65	25.8 ± 0.95
2	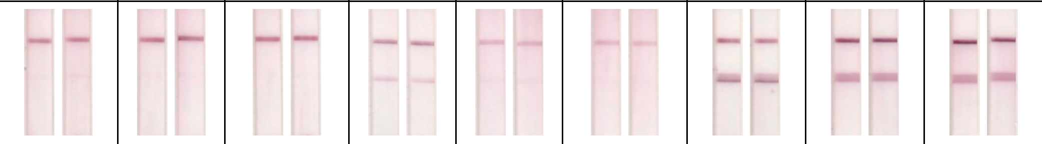								
I (a.u.)	2.9 ± 0	2.0 ± 0.3	1.78 ± 0.35	10.1 ± 0.6	3.5 ± 0.35	0	29.2 ± 0.65	26.9 ± 0.6	25.4 ± 0.1
3	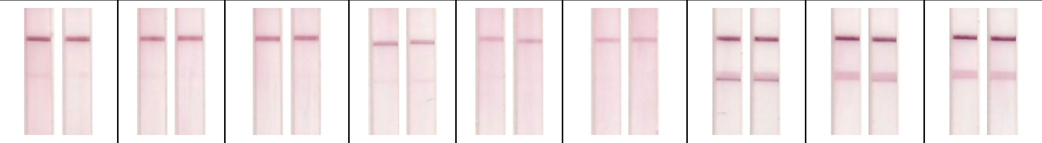								
I (a.u.)	4.8 ± 0.08	2.1 ±0.15	0	3.6 ± 0.28	2.5 ± 0.03	0	21.5 ± 0.45	17.6 ± 0.1	14.9 ± 0.33
4	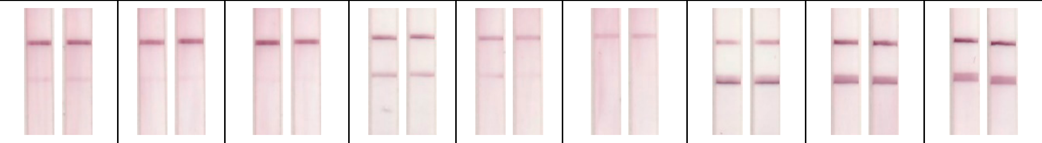								
I (a.u.)	5.9 ± 0.35	2.9 ± 0.03	0	17.5 ± 0.4	4.6 ± 1.15	0	34.6 ± 0.25	33.9 ± 1.15	28.1 ± 0.8
5	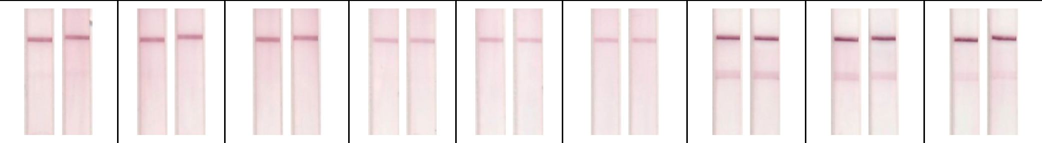								
I (a.u.)	2.9 ± 0.5	0	0	0	0	0	11.6 ± 0.95	9.7 ± 0.56	5.1 ± 0.2
6	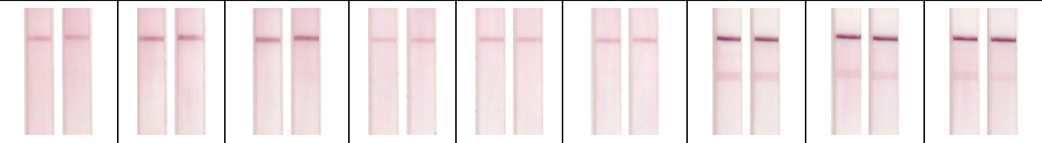								
I (a.u.)	0	0	0	0	0	0	11.0 ± 0.3	7.5 ± 0.25	5.9 ± 0.85
7	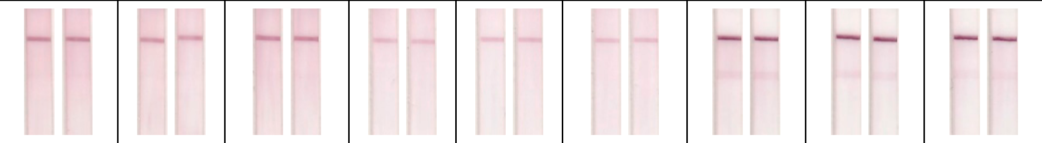								
I (a.u.)	2.2 ± 0.25	0	0	0	0	0	5.8 ± 0.5	5.1 ± 0.38	3.0 ± 0.03

8	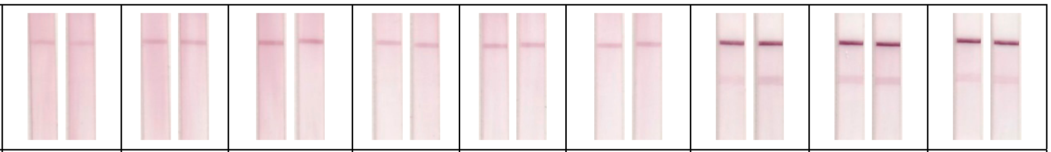								
I (a.u.)	1.4 ± 0.15	0	0	0	0	0	10.3 ± 1.17	8.8 ± 1.38	6.6 ± 0.45
9	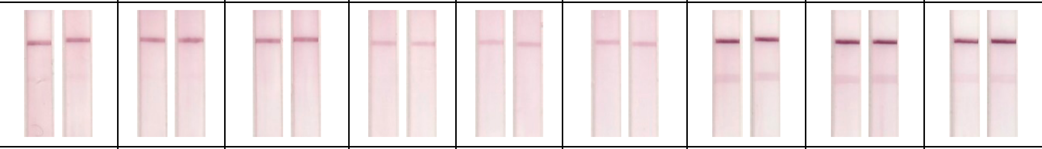								
I (a.u.)	3.3 ± 0.6	2.0 ± 0.58	0	0	0	0	8.5 ± 0.5	8.8 ± 0.2	4.9 ± 0.35
10	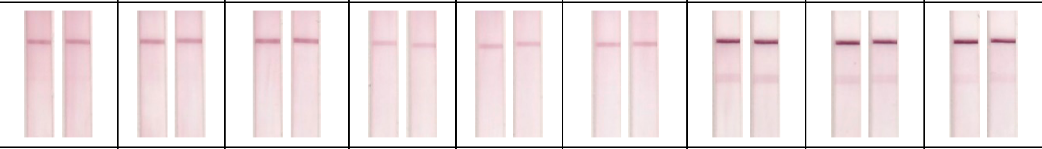								
I (a.u.)	2.2 ± 0.43	0	0	0	0	0	8.7 ± 0.25	7.4 ± 0.25	4.2 ± 0.5
11	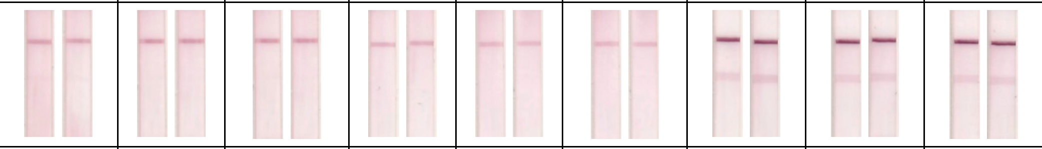								
I (a.u.)	2.2 ± 0.2	0	0	0	0	0	10.4 ± 0.9	8.3 ± 0.28	6.4 ± 0.25
12	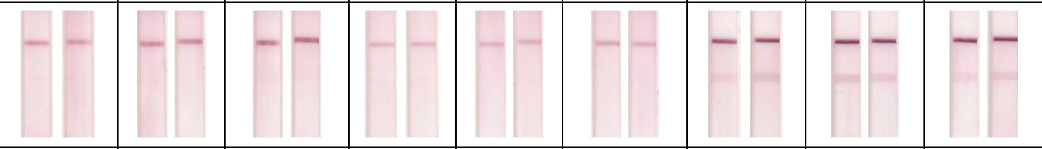								
I (a.u.)	1.6 ± 0.14	0	0	0	0	0	9.6 ± 1.4	11.6 ± 0.4	8.7 ± 1.13
13	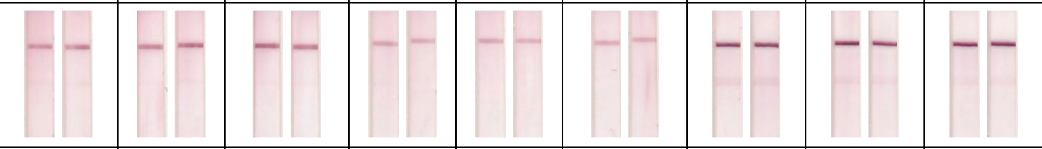								
I (a.u.)	2.7 ± 0.4	2.0 ± 0.5	0	1.6 ± 0.1	0	0	5.6 ± 0.05	4.1 ± 0.55	3.0 ± 0.3
14	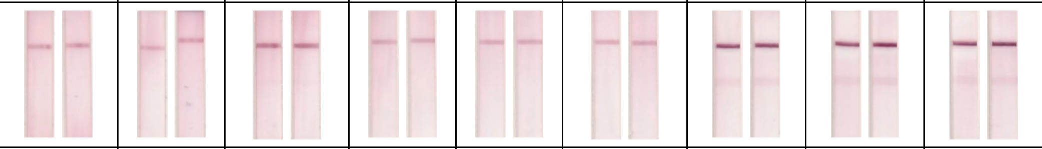								
I (a.u.)	0	0	0	0	0	0	5.4 ± 0.1	6.4 ± 0.2	5.6 ± 0.4
15	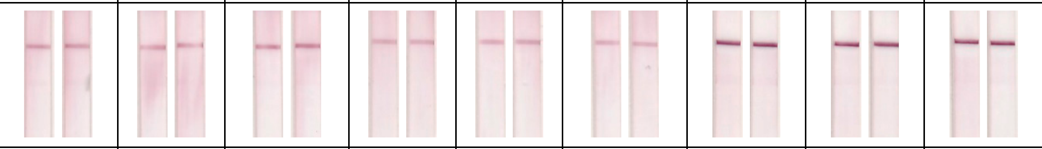								
I (a.u.)	0	0	0	0	0	0	1.6 ± 0.29	0	0

16	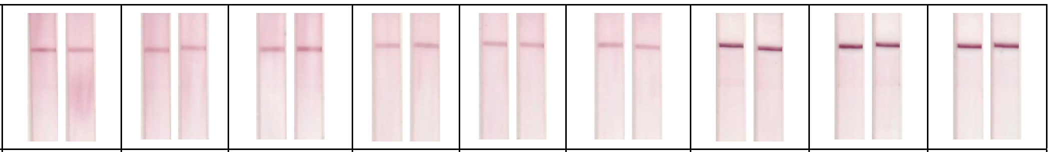								
I (a.u.)	0	0	0	0	0	0	2.1 ± 0	0	0
17	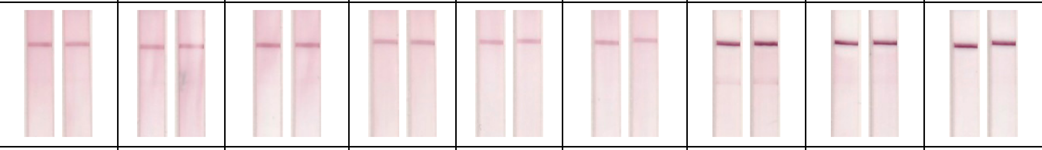								
I (a.u.)	0	0	0	0	0	0	3.9 ± 0.03	1.8 ± 0.5	0
18	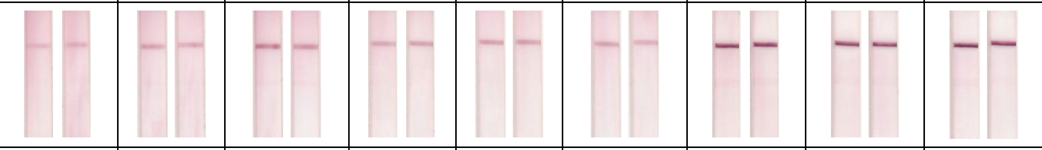								
I (a.u.)	0	0	0	0	0	0	2.7 ± 1.3	0	0
19	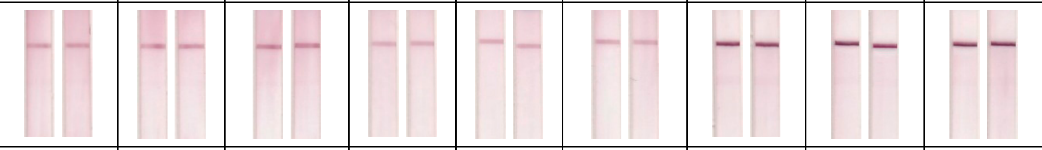								
I (a.u.)	0	0	0	0	0	0	2.6 ± 0.05	0	0
20	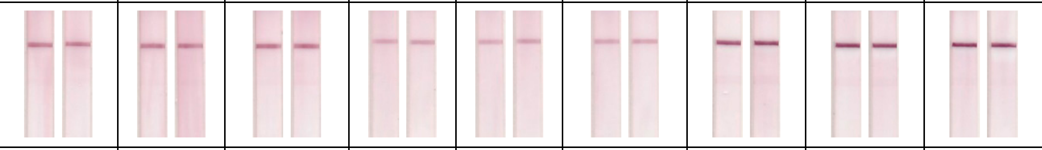								
I (a.u.)	0	0	0	0	0	0	3.5 ± 0.3	0	0
21	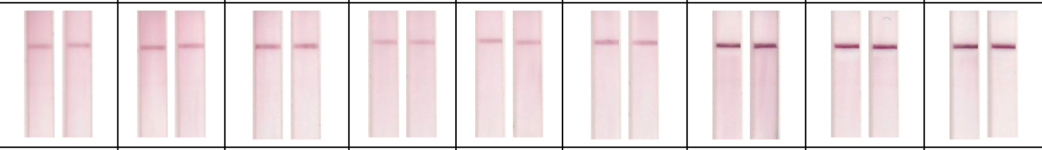								
I (a.u.)	0	0	0	0	0	0	0	0	0
22	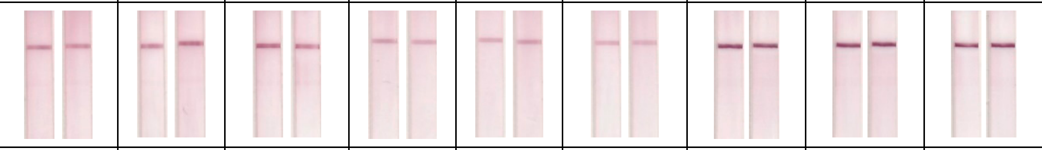								
I (a.u.)	0	0	0	0	0	0	0	0	0
